# Detection and characterization of carbapenem resistant Gram‐negative bacilli isolates recovered from hospitalized patients at Soba University Hospital, Sudan

**DOI:** 10.1186/s12866-021-02133-1

**Published:** 2021-05-04

**Authors:** Hana S. Elbadawi, Kamal M. Elhag, Elsheikh Mahgoub, Hisham N. Altayb, Francine Ntoumi, Linzy Elton, Timothy D. McHugh, John Tembo, Giuseppe Ippolito, Abdinasir Yusuf Osman, Alimuddin Zumla, Muzamil M. Abdel Hamid

**Affiliations:** 1 Institute of Endemic Diseases, University of Khartoum, Khartoum, Sudan; 2 Soba University Hospital, University of Khartoum, Khartoum, Sudan; 3 Ahfad University for Women, Omdurman, Sudan; 4 Department of Microbiology, Faculty of Medicine, University of Khartoum, Khartoum, Sudan; 5 Department of Biochemistry, College of Sciences, King Abdul-Aziz University, Jeddah, Kingdom of Saudi Arabia; 6 Université Marien NGouabi, Fondation Congolaise pour la Recherche Médicale (FCRM), Brazzaville, Congo; 7 Institute for Tropical Medicine, University of Tübingen, Tübingen, Germany; 8 Center for Clinical Microbiology, University College London, Royal Free Campus, Rowland Hill Street, NW3 2PF London, United Kingdom; 9 UNZA-UCLMS and HERPEZ Research and training programs, University teaching Hospital, Lusaka, Zambia; 10 National Institute for Infectious Diseases, IRCCS, Via Portuense 292, 00149 Lazzaro Spallanzani, Rome, Italy; 11 The Royal Veterinary College, University of London, Hawkshead Lane, North Mymms, Hatfield, Hertfordshire UK; 12 UCL Hospitals NIHR Biomedical Research Centre London, London, UK

**Keywords:** Hospitalized patients, Carbapenemase resistant genes, Multidrug‐resistant, Gram negative bacteria, Sudan

## Abstract

**Background:**

Antimicrobial resistance (AMR) poses a complex threat to global health security and universal health coverage. Recently, nosocomial infections with carbapenemase-producing Gram-negative bacilli (GNB) is increasing worldwide. We report the molecular characterization and detection of genes associated with carbapenemase producing Gram negative bacteria isolated from hospitalized patients at Soba University Hospital (SUH) in Khartoum State, Sudan.

**Results:**

Between October 2016 and February 2017, a total of 206 GNB clinical specimens were collected from hospitalized patients in SUH. Of 206 carbapenem resistance isolates, 171 (83 %) were confirmed as phenotypically resistant and 121 (58.7 %) isolates harboured one or more carbapenemase genes. New Delhi metallo-β-lactamase (NDM) types were the most predominant genes, *bla*NDM 107(52 %), followed by *bla*IMP 7 (3.4 %), *bla*OXA-48 5(2.4 %) and *bla*VIM 2 (0.9 %). Co-resistance genes with NDM producing GNB were detected in 87 (81.3 %) of all *bla*NDM producing isolates. NDM-1 was the most frequent subtype observed in 75 (70 %) *bla*NDM producing isolates. The highest percentage of resistance was recorded in ampicillin (98 %), cephalexin (93.5 %) amoxicillin clavulanic acid (90 %), cefotaxime (89.7 %), ceftriaxone (88.4 %), ceftazidime (84.2 %), sulfamethoxazole-trimethoprim (78.4 %) and nitrofurantoin (75.2 %), aztreonam (66 %) and temocillin (64 %). A close correlation between phenotypic and carbapenemase genes detection in all GNB was observed.

**Conclusions:**

The frequency of carbapenemase producing bacilli was found to be high in SUH. NDM was found to be the most prevalent carbapenemase gene among clinical isolates. Close surveillance across all hospitals in Sudan is required. The relative distribution of carbapenemase genes among GNB in nosocomial infections in Africa needs to be defined.

## Background

Antimicrobial resistance (AMR) poses a complex threat to global health security and universal health coverage. The prevalence and distribution of antimicrobial resistant bacterial infections in the nosocomial settings in Africa is poorly defined [1, 2]. Carbapenems have been considered as a robust group of antibiotics to treat extended spectrum β-lactamase (ESBL)- producing bacteria in the past ten years and are widely prescribed for treatment of multidrug-resistant Gram-negative bacilli in systemic infections [3]. Overuse of these drugs can favor the selection and spread of multidrug resistant bacteria as well as Carbapenem resistant Enterobacteriales (CRE) [4] CRE, carbapenem-resistant *Pseudomonas aeruginosa*, and carbapenem-resistant *Acinetobacter baumannii* were ranked as ‘critical’ and ‘high’ priority pathogens by the World Health Organization (WHO) in 2017 [5].

Carbapenem resistance is associated with the production of carbapenemase, encoded by what is termed carbapenemase encoding genes, and categorized as four classes of ß-lactamase, including class A carbapenemases such as *Klebsiella pneumoniae* carbapenemases (*bla*KPC), imipenem- hydrolyzing β-lactamase (*bla*IMI) and *Serratia marcescens* enzyme (*bla*SME). Class B Metallo-beta-lactamases such as New Delhi Metallo-beta-lactamases (*bla*NDM), verona integron metallo-beta-lactamases (*bla*VIM), imipenemase (*bla*IMP), German imipenemase (*bla* GIM-1) and Sao Paulo Metallo-beta-lactamases (*bla*SPM). While class D carbapenemases including oxacillinase-group (*bla*OXA-48, OXA- 181, OXA-204, OXA-162, OXA-23, OXA-24) and a rare class C ß-lactamase, including cephamycin-hydrolyzing β-lactamase (*bla*CMY-10) [6, 7]. Resistance to carbapenems can occur by other mechanisms including overproduction of ESBLs or AmpC enzyme in combination with porin mutations, which reduces outer membrane permeability, and activation of multidrug efflux pumps in response to antibiotic exposure [8]. Plasmids encoding carbapenemases may also carry co-resistance genes for resistance to other β-lactam and non β-lactam antibiotics [7]. There is a significant challenge in controlling the spread of carbapenemases. Surveys of the molecular epidemiology of carbapenemases revealed that the dissemination of carbapenemases, including NDM, VIM, IMP, OXA-48 and KPC producers, are rapid and widespread among healthcare facilities [9, 10].

Detection of carbapenemase producing isolates by clinical microbiology laboratories is essential to provide targeted therapy, antimicrobial stewardship and update local antibiotic guidelines for clinicians. Furthermore, screening of resistance mechanisms using of antimicrobial susceptibility test in addition to detection of genes associated by phenotype and molecular analysis provides confirmation of clinically observed treatment failure. Whilst over the past decade, there has been an increase in reports globally of nosocomial infections caused by carbapenem resistant Gram-negative bacilli (GNB), data from Africa have been scanty and antimicrobial stewardship is not optimally practiced. This study aimed to detect and characterize carbapenem resistance GNB isolated from patients treated at Soba University Hospital in Khartoum state, Sudan.

## Results

### Demographic distribution

**Fig. 1:**
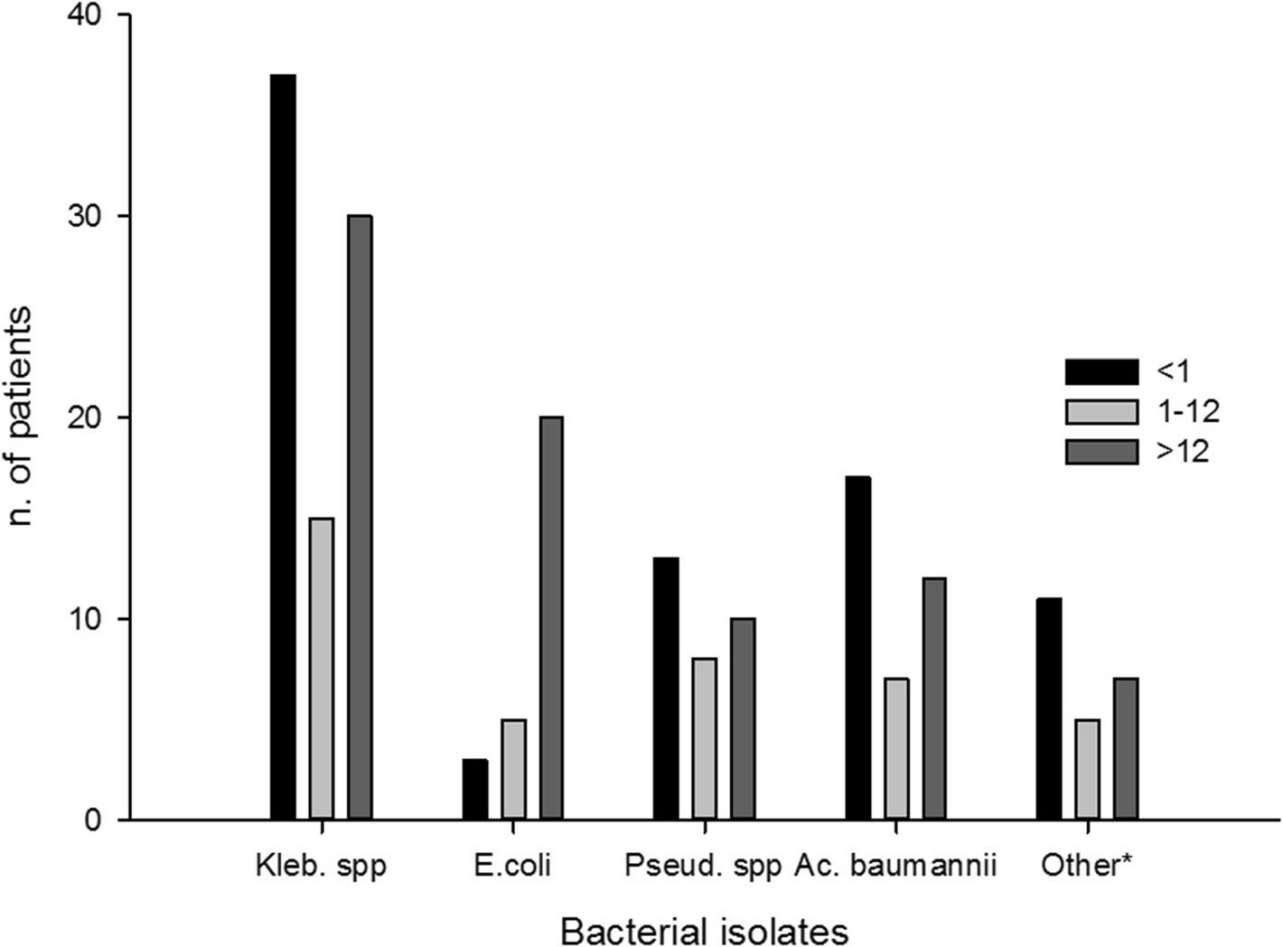
Distribution of bacterial species isolates among different age group

The demographic characteristics of the inpatients and the frequency of GNB isolates according to age groups are shown in Fig. 1. Most of the isolates were from pediatric patients less than one year old (42.5 %), followed by age group ranged from 13 to 80 years (38 %) and the rest of pediatric patients age group ranged from 1 to 12 years (19.5 %). Males 53.4 % (110/206) were predominant among inpatients, with females at 46.6 % (96/206). With regard to the distribution of carbapenemase producers by hospital location, the most carbapenemase producing isolates were found in the Neonatal Intensive Care Unit 32(26 %), followed by Medicine wards 26(22 %), Pediatric wards 22 (18 %), Surgery 18(15 %), ICU 15(12 %) and the Renal Unit 8(7 %). Carbapenemase producing isolates were most frequently distributed among the following clinical specimens; blood (36 %) followed by wound samples (24 %), urine (21 %), body fluids (7 %), catheter tips (6 %) and sputum samples (6 %).

### Antimicrobial susceptibility and phenotypic screening

**Fig. 2:**
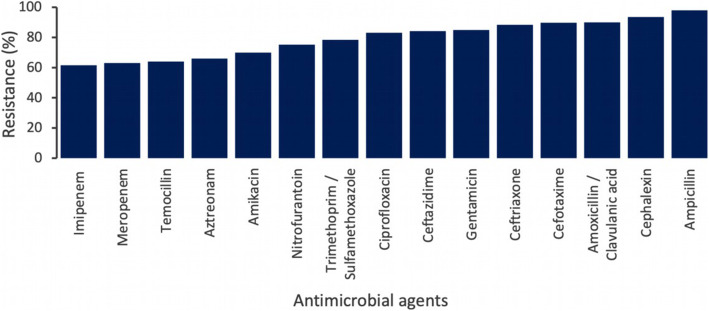
Antimicrobial Resistance pattern among different Gram-negative bacilli isolated from patients treated at Khartoum state hospitals

The antibiotic resistance pattern is shown in Fig. 2. Out of 206 isolates tested, the highest percentage of resistance was recorded in ampicillin (98 %) and cephalexin (93.5 %), amoxicillin clavulanic acid (90 %), cefotaxime (89.7 %), ceftriaxone (88.4 %), ceftazidime (84.2 %), sulfamethoxazole-trimethoprim (78.4 %) and nitrofurantoin (75.2 %), aztreonam (66 %) and temocillin (64 %). The resistance rate was also high in ciprofloxacin (83.1 %), gentamicin (85 %) and amikacin (70 %). The resistance rate for meropenem and imipenem was 63.1 % and 61.6 %, respectively.

Phenotypic carbapenemase activity was detected in 171 (83 %) of the 206 clinical isolates. These isolates were positive for the production of one or more carbapenemase enzymes. Details of the carbapenemase activity among different isolates and phenotypic tests are given in Table 1.

**Table 1:** Frequency of carbapenemase producing among Gram-negative bacilli by phenotypic tests

	Positive isolates for a particular phenotypic test				Total
Bacterial isolates	EDTA	MHT + BA	EDTA + BA	EDTA + AB + MHT	
	n (%)	n (%)	n (%)	n (%)	n (%)
*E.coli* (*n* = 28)	17 (16.4)	2 (8.3)	3 (11.1)	4 (26.6)	26 (15.2)
*Klebsiella spp*. (*n* = 82*)*	41 (39)	10 (41.6)	12 (44.4)	8 (53.3)	71 (41.5)
*Pseudomonas spp.* (*n* = 45)	19 (18)	9 (37.5)^a^	4 (14.9)	1 (6.6)	33(19.2)
*Acin. baumannii* (*n* = 36)	19 (18)	3 (12.6) ^a^	6 (22.2)	2 (13.3)	30 (17.6)
*Burkholderia cepacia* (*n* = 2)	2 (1.9)	0 (0)	0 (0)	0 (0)	2 (1.2)
*Enterobacter spp.* (2)	2 (1.9)	0 (0)	0 (0)	0 (0)	2 (1.2)
*Other GNB*^b^ (*n* = 11)	5 (4.8)	0 (0)	2 (7.4)	0(0)	7 (4)
Total (*n* = 206)	105(50.9)	24 (11.7)	27 (13.1)	15 (7.2)	171(83)

-MHT positive = KPC + OXA48 - Boronic acid positive = KPC

-MHT + Boronic acid positive = KPC -MHT positive + boronic acid negative = OXA 48

Note: MHT applied only for Enterobacterceae. -EDTA positive = Metallo-β-lactamase^.a^These isolates just positive by BA and not MHT.^b^Other Gram-negative bacilli include: Citrobacter species (3), Serratia species (1), Proteus spp. (2), Stenotrophomonas maltophilia (3), Vibrio vurneficus (1) and Morganella morganii (1).

### Prevalence and distribution of carbapenemase genes among Gram negative bacilli

One or more carbapenemase genes were detected in 121 (58.7 %) of the 206 study isolates using PCR. *bla*NDM was the most commonly detected among the isolates, predominantly in *K. pneumonia*. *bla*NDM was also detected more often in *A. baumannii*, *P. aeruginosa* and *E. coli*. The most prevalent gene was *bla*NDM 107(52 %), followed by *bla*IMP 7 (3.4 %), *bla*OXA-48 5(2.4 %), *bla*VIM 2 (0.9 %) and *bla*KPC 0 (0 %). ESBLs were detected among these isolates with high prevalence in 183 isolates (88.8 %), with the following genes: *bla*CTXM 126(61.6 %), *bla*SHV 84(40.7 %) and *bla*TEM 78(37.8). The genes were unevenly distributed among the different study isolates and more details are given in Table 2.

**Table 2:** Distributions of carbapenemase and ESBL genes among GNB isolates

Bacterial isolates	Carbapenemase genes					ESBL genes		
	NDM	OXA-48	IMP	VIM	KPC	CTXM	SHV	TEM
	n(%)	n (%)	n (%)	n (%)	n (%)	n (%)	n (%)	n (%)
*K. pneumoniae* (*n* = 82)	58 (54.3)	1 (20)	3 (43)	1 (50)	0 (0)	53 (41.7)	28 (33.4)	32 (40)
*E.coli* (*n* = 28)	9 (8.4)	1 (20)	2 (28.5)	0 (0)	0 (0)	14 (11.6)	6 (7.2)	4 (5)
*Pseudomonas Spp*.(*n* = 45)	14 (13.1)	2 (40)	2 (28.5)	1 (50)	0 (0)	28 (22.3)	18 (21.5)	16 (20)
*A. baumannii* (*n* = 36)	17 (15.8)	1 (20)	0 (0)	0 (0)	0 (0)	20 (15.7)	24 (28.6)	18 (22.5)
*Burkholderia* (*n* = 2)	0	0 (0)	0 (0)	0 (0)	0 (0)	2 (1.6)	2 (2.4)	4 (5)
*Enterobacter Spp.*(*n* = 2)	2 (1.8)	0 (0)	0 (0)	0 (0)	0 (0)	2 (1.6)	1 (1.2)	1 (1.25)
Other GNB^a^ (*n* = 11)	7 (6.5)	0 (0)	0 (0)	0 (0)	0 (0)	7 (5.5)	4 (5.7)	5 (6.25)
Total (*n *= 206)	107 (52)	5 (2.4)	7 (3.4)	2 (0.9)	0 (0)	126 (61.1)	84 (40.7)	78 (37.8)

^a^Other Gram-negative bacilli include: *Citrobacter* species (3), *Serratia* species (1), *Proteus spp.* (2), *Stenotrophomonas maltophilia* (3), *Vibrio vurneficus* (1) and *Morganella morganii* (1)

Co-resistance carbapenemase genes were observed in a small number of isolates; *bla*NDM + *bla*OXA-48 were detected in three isolates, while *bla*NDM + *bla*VIM and *bla*NDM + *bla*IMP were detected in two different isolates.

Out of 107 *bla*NDM genes detected, 75 (70 %) were *bla*NDM-1. Other subtypes of *bla*NDM were identified by sequencing, including *bla*NDM- 5, and *bla*NDM-6. The sequence of the 14 NDM genes was analyzed to confirm the presumed most prevalent (NDM) gene type and all showed 97–100 % similarity with *bla*NDM genes from the NCBI database with accession number MF379688 and MG764089. Thereafter, all sequences have been deposited in the GenBank database under the following accession numbers: MK033562, MK033563, MK033564, MK363705, MK363706, MK363707, MK363708, MK363709, MK363710, MK371542, MK371543, MK371544, MK37154 5, and MK371546.

Our analysis revealed that phenotypic test versus Carbapenemase or ESBL gene detection were strongly correlated (*P* = 0.0000001; *P* = 0.01, respectively). The correlation between phenotypic and carbapenemase genes detection was highly significant for *K. pneumonia, E. coli, P. aerugnosa* and *A. baumannii* (*P* = 0.0000031; *P* = 0.00079; *P* = 0.015; *P* = 0.02, respectively) while *for* ESBL genes correlation was only significant for *P. aerugnosa* (*P* = 0.038) Table 3.

**Table 3:** Comparison between phenotypic and genotypic results of carbapenem resistance among different GNB strains

Bacterial species	Resistant isolates n	Phenotypic test^a^ n (%)		Carbapenemase genes^b^ n (%)		Correlation with carbapenemase	ESBL genes^c^ n (%)		Correlation with ESBL
		positive	negative	positive	negative	*p-*value	positive	negative	*p-*value
*K. pneumoniae*	82	71 (86.6)	11(13.4)	63 (76.8)	19 (23.2)	0.0000031	72 (87.8)	10 (12.2)	0.017
*E.coli*	28	26 (92.8)	2 (7.2)	12 (42.8)	16 (57.2)	0.00079	19 (67.8)	9 (32.2)	0.123
*P. aeruginosa*	45	33 (73)	12 (27)	19 (42)	26 (58)	0.0015	39 (86.6)	6 (13.4)	0.038
*A. baumannii*	36	30 (83.3)	6 (16.7)	18 (50)	18 (50)	0.022	31 (86)	5 (14)	0.14
Other GNB^d^	15	11 (73)	4 (27)	9 (60)	6 (40)	0.055	15 (100)	0 (0)	0.08
Total	206	171 (83)	35 (17)	121(58.7)	85 (41.3)	0.0000001	176 (85.4)	30 (14.6)	0.01

^a^Phenotypic test include: EDTA, Borinic Acid and Modified Hodge Test

^b^Carbapenemase genes include: NDM, VIM, IMP and OXA-48

^c^ESBL genes include: CTX-M, SHV, and TEM

^d^Other GNB include: *Citrobacter species* (3), *Burkholderia spp.* (2), *Enterobacter Spp.*(2), *Serratia species* (1), *Proteus spp.* (2), *Stenotrophomonas maltophilia* (3), *Vibrio vurneficus (1), and Morganella morganii* (1)

### Co‐resistance genes carried with NDM producing gram‐negative bacilli

Several isolates carried more than one type of gene including *bla*NDM. ESBL were often observed together with *bla*NDM in 87 (81.3 %) of *bla*NDM producing isolates (107). Most of the isolates carried *bla*NDM with one ESBL gene in 38(43.5 %) as the following: *bla*NDM + *bla*CTXM in (24 isolates, 27.6 %), *bla*NDM + *bla*TEM (8 isolates, 9.1 %), and *bla*NDM + *bla*SHV (6 isolates, 6.8 %). Isolates carried *bla*NDM with two ESBL genes in (39.2 %) as the following: *bla*NDM + *bla*CTXM + *bla*SHV (10 isolates, 11.5 %), *bla*NDM + *bla*CTXM + *bla*TEM (10 isolates, 11.5 %), *bla*NDM + *bla*SHV + *bla*TEM (14 isolates, 16.2 %). Isolates carried *bla*NDM with three ESBL genes, *bla*NDM + *bla*CTXM + *bla*SHV + *bla*TEM in 15 isolates (17.3 %). The distribution of co-resistance genes among different Gram-negative bacilli is shown in Table 4.

**Table 4:** Co resistance genes with *bla*NDM among Gram-negative bacilli

*Bla genes*	*K. pn*	*E.coli*	*P. aer*	*A. bau*	*Enter*	Total (%)
*NDM + CTXM*	15	2	5	2	0	24 (27.6 %)
*NDM + SHV*	2	0	2	2	0	6 (6.8 %)
*NDM + TEM*	4	1	1	1	**1**	8 (9.1 %)
*NDM + CTXM + SHV*	7	0	1	1	1	10 (11.5 %)
*NDM + CTXM + TEM*	6	2	0	2	0	10 (11.5 %)
*NDM + SHV + TEM*	7	0	1	6	0	14 (16.2 %)
*NDM +* All	9	1	3	2	2	15 (17.3 %)
Total	50	6	13	16	2	87 (100 %)

- *K. pn = Klebsiella pneumoniae, P. aer = Pseudomonasa aeruginosa*, *A. bau = Acinetobacter baumannii, Enter = Enterobacter spp*

## Discussion

Carbapenems have become the drug of choice for the treatment of severe nosocomial infections caused by Gram-negative bacilli. Carbapenem resistant Enterobacteriales (CRE) is a considerable health problem globally and are associated with increased mortality [3], and thus rapid detection of carbapenem resistance and adequate treatment of such cases is mandatory in health care settings. This study was undertaken to determine the prevalence of different types of carbapenemase producing bacteria among Gram-negative bacilli isolated from various hospitalised patients at Soba University Hospital. The accurate detection of carbapenemase producing microorganisms is challenging for laboratories and requires phenotypic and genotypic analysis. Of 206 isolates, 171(83 %) were positive by phenotypic analysis, including isolates with resistance to carbapenems. The genotypic analysis detected 121 (58.7 %) positive isolates.

Our results revealed that the prevalence of carbapenemase production among different Gram-negative isolates is increasing (up to 83 %). Of note, this finding is higher than the incidence observed in a previous study conducted in Khartoum State in 2017, which showed a prevalence of 56 % by phenotypic tests (unpublished data). The high frequency of MBL in Khartoum State might be attributed to the excessive use of meropenem in the treatment of patients associated with ESBL infections. Our results are in agreement with a study in Egypt, which reported that carbapenem resistance was 62.7 % among Enterobacterial [12]. High rates of carbapenem resistance in 28.6 % of isolates have also been observed in Uganda by Okoche in 2015 [13]. In Tanzania, the prevalence of carbapenemase producing isolates was 35 % [14]. In South Africa, it was found to be 68 % [15], and in Nigeria, 11.9 % [16]. Carbapenem resistance in low and middle-income countries (LMICs) in Africa is likely to increase as a result of unrestricted usage of antibiotics, as the majority of the population consumes antibiotics without a clinical prescription [17].

Carbapenemase genes have been recently recognized, and these genes are associated with mobile genetic elements that allow their rapid circulation among bacterial isolates. For instance, *bla*NDM has the potential for rapid spread, as evidenced in Turkey and other countries across the globe [18]. In this study, carbapenemase genes were detected using PCR in 121 (58.7 %) of the sampled isolates. The most prevalent gene among the isolates was *bla*NDM (88.4 %), mainly in *K. pneumonia* and other Gram-negative bacilli, including *A. baumannii*, *P. aeruginosa* and *E. coli*. Our results are comparable with those reported in India, South Africa, Saudi Arabia and other Middle Eastern countries [15, 19–21].

Carbapenemase genes are reported to be more frequent in some regions. For example, *bla*KPC genes are dominant in some countries such as Greece, Israel, and USA, while *bla*NDM genes are prevalent in isolates reported from the Far East, India, and Pakistan [11]. OXA-48 was first reported from Turkey, followed by reports from Central Asia and Europe [22]. In the current study, the genes were unevenly distributed among the different bacterial isolates. The *bla*NDM gene was found in high prevalence (52 %) compared to other genes. Our findings, however, differ with several studies (for instance, in the Okoche study, the most common gene was *bla*VIM (10.7 %), and *bla*NDM-1 (2.6 %) was the lowest gene [13], while Mushi reported IMP types were the most predominant at 21.6 % in their study [14]. Other studies reported that *bla*OXA-48 was the most prevalent gene [23, 24]. In contrast to global reports of a high prevalence of *bla*KPC genes [11, 25], we have not detected *bla*KPC among the tested isolates. NDM variants have been described differing by several amino acid changes include, *bla*NDM-2, *bla*NDM-4, *bla*NDM-5, *bla*NDM-6 and *bla*NDM-7 [26]. In this study, 107 *bla*NDM producer isolates had been identified using PCR, the most common subtype 75 (70 %) was *bla*NDM-1. Other subtypes of *bla*NDM were detected by sequencing including *bla*NDM-5, and *bla*NDM-6 among different Gram-negative bacilli, including *K. pneumoniae, E. coli, A. baumannii, P. aeruginosa* and *Enterobacter spp.*

Carbapenemase producers are becoming highly distributed among Enterobacterales, *A. baumannii*, *P. aeruginosa* and other Gram-negative bacilli. The highest prevalence of carbapenemase production in this study was observed in *K. pneumoniae*, followed by *P. aeruginosa*, *A. baumannii* and *E. coli* Table 1. Similar studies in which *K. pneumonia, A. baumannii* and *Pseudomonas spp.* as the most common carbapenemase producing isolates were reported [27–29]. The prevalence of carbapenemase producing isolates varies across hospital settings. This variation could be attributed to a wide range of factors including differences in collection time of isolates, study designs and target populations. Common carbapenemase encoding genes have been associated with bacteria isolated from blood, urine, wounds and sputum, as reported in many studies in Africa and India [13, 14, 16, 30]. In this finding, carbapenem producers were more frequently isolated from blood followed by wounds and urine as these results are compatible with a study in South Africa in which blood was the most common specimen type (25 %), followed by urine (22 %) [15].

Young patient age has long been considered as a risk factor for Carbapenem resistant Enterobacteriales (CRE) infection, which agrees with this study’s finding that carbapenemase-producing Gram negative bacilli were most frequent in children less than one year of age, located in the neonatal and pediatric wards. High rates of carbapenem resistant infections were observed among elderly patients from Medicine wards (22 %) and ICU (12 %), which agrees with another study that found CRE to be more frequently isolated in the elderly [31].

Carbapenem resistant Gram-negative bacilli are usually resistant to other routinely used antimicrobial agents [32–34]. The plasmids carrying carbapenemase genes like *bla*NDM-1 are diverse and can harbor a high number of additional resistance genes (e.g., ESBL-alleles) as well as other carbapenemase genes like *bla*OXA-48, *bla*VIM. These plasmids were considered as the source of multidrug resistance in one single bacterium [20, 35]. Moreover, mechanisms of resistance to β-lactam antibiotics via the production of ESBL, AmpC and carbapenemase were also noticed among the isolates that produce different combinations of the enzymes. In the present study, co-resistance of *bla*NDM with *bla*OXA-48, *bla*VIM and *bla*IMP were reported in few isolates. In connection to co-resistance with ESBL, *bla*CTXM, *bla*SHV and *bla*TEM were detected in a high prevalence of *bla*NDM positive isolates. Most of the isolates carried *bla*NDM with one or more ESBL genes. This is in agreement with various studies that have reported co-resistance among clinical isolates [36, 37]. These multiple resistance genes found in some isolates, as observed in this study, are indicative of the existence of multidrug resistant pathogens, which are responsible for treatment failure, outbreaks of infections and higher treatment costs [38].

Sudan is a large country that shares its borders with seven other countries. People move freely between these borders with the potential passage of antibiotic resistant strains. The dynamic movements of people will make it challenging to monitor AMR in these countries, especially at the borders. These challenges may also represent an opportunity for wider continental monitoring and collaboration between countries rather than country-specific. Such an approach will aid in universal and intergovernmental initiatives to control and limit the AMR spread.

## Conclusions

The frequency of carbapenemase producing bacilli was found to be high in Soba University Hospital (SUH). *bla*NDM was found to be the most prevalent carbapenemase gene among clinical isolates. Improved antibiotic stewardship and infection control measures and close surveillance across all hospitals in Sudan is required. The relative distribution of carbapenemase genes among Gram-negative bacilli (GNB) in nosocomial infections in Africa needs to be investigated.

## Methods

### Study design and clinical isolates

A cross-sectional laboratory-based study was conducted at the microbiology department in Soba University Hospital and Unit of Molecular Biology, Institute of Endemic Diseases (IEND), University of Khartoum; involving Gram negative clinical bacterial isolates, suspected as carbapenemase producing based on breakpoints zone diameter of carbapenems (CLSI, 2017) [39]. These were isolated from cultures of various clinical specimens; blood, urine, wound swabs, sputum, tips of catheters and other body fluids, between 1st October 2016 and 25th February 2017 from inpatients at Soba University Hospital. Quality control strains [*E*. *coli* (ATCC #25,922) and *P. aeruginosa* (ATCC #27,853)] were used in antimicrobial susceptibility testing. Standard biochemical tests were used for primary identification [40] and molecular identification using PCR [8] was used for all study isolates with universal primer (16SrRNA). For species-specific isolates identified on biochemical testing, species-specific primers for *Klebsiella pneumoniae*, *Escherichia coli*, *Pseudomonas aeruginosa* and *Acinetobacter baumannii* were used for confirmation [41–52]. All isolates were stored in 20 % glycerol at -20°C until use.

### Subculture, susceptibility testing, phenotypic screening and confirmatory tests for carbapenemase resistance

Selection of antimicrobial panels and interpretation of disk diffusion for each bacteria was completed according to the Clinical and Laboratory Standards Institute (CLSI) guidelines [39].

Isolates were subcultured on blood agar (BA) and then subjected to susceptibility testing with the following antimicrobials (Mast Diagnostic, UK): amoxicillin-clavulanate (AMC) (30µg); cefuroxime (CXM) (30µg); cephalexin (CL) (30µg); ceftriaxone (CRO) (30µg); ceftazidime (CAZ) (30µg); cefotaxime (CTX) (30µg); meropenem (MEM) (10µg); imipenem (IPM) (10µg); amikacin (AK)(30µg); gentamicin (CN)(10µg); ciprofloxacin (CIP)(5µg); trimethoprim-sulfamethoxazole (SXT)(25µg); temocillin (TEM)(30µg); aztreonam (AZT)(30µg). The Kirby Bauer(disk diffusion) was performed; each isolate was swabbed onto Muller-Hinton agar and the antibiotic discs were placed on top, incubated at 37 °C for 18–24 hours [39, 40].

Bacterial isolates that showed intermediate or resistance to imipenem or meropenem were considered as suspected carbapenemase producers and were screened. Phenotypic confirmatory tests for carbapenemase production were conducted using the boronic acid synergy test for class A β-lactamases, the EDTA synergy test for metallo-β-lactamase and the Modified Hodge Test (MHT) for Enterobacterales to detect KPC and OXA-48 producers in addition to temocillin sensitivity [46].

### Molecular identification of carbapenemase encoding genes

PCR was carried out using a thermal cycler and the following primers (Macrogen, Korea), *bla*VIM, *bla*IMP, *bla*NDM, *bla*NDM-1, *bla*KPC, *bla*OXA-48, *bla*TEM, *bla*SHV and *bla*CTX-M genes were used [47–51]. The reaction was carried out in a total reaction volume of 25µl (5µl master mix, Maxime RT premix kit) [8]. The purity and integrity of each PCR product was evaluated, and the amplified product was confirmed with reference to the standard DNA ladder.

### DNA sequencing and genes analysis

The PCR product of *bla*NDM genes and 16SrRNA were purified and Sanger sequencing was performed by Macrogen Company (*Seoul, Korea*). Fourteen *bla*NDM gene products have been selected randomly to represent the study isolates.

### Bioinformatics analysis

Firstly, DNA sequences were clarified and determined the overall quality of the sequences by reviewing nucleotide chromatogram by using Finch TV software version 1.4.0 () to ensure the ambiguous sites. Thereafter, nucleotide sequences of the NDM genes identified were searched for sequence similarity using nucleotide BLAST [52] (http://blast.ncbi.nlm.nih.gov/Blast.cgi).

### Statistical analysis

Data were analysed using SPSS software version 20.0. Cross tabulation was used to present the relationships between data of antimicrobial sensitivity, phenotypic tests and resistant gene detection among the study isolates, qualitative data were performed through χ^2^ test and significance was set at *p ≤* 0.05.

## Data Availability

The antimicrobial data datasets used and/or analyzed during the current study are available at . blaNDM gene sequences are deposited at NCBI under the accession numbers: MK033562- MK033564, MK363705-MK363710 and MK371542- MK371546 for. The 16S rRNA sequences generated in this study are available from GenBank under sequential accession numbers MW034199–MW034227.

## Abbreviations

AK: Amikacin
AMC: Amoxicillin clavulanate
Amp C: Class C β-lactamases
AMR: Antimicrobial resistance
ATCC: American Type Culture Collection
AZT: Azteroname
BLAST: Basic local alignment search tool
bp: Base pair
CAZ: Ceftazidime
CIP: Ciprofloxacin
CL: Cephalexin
CLSI: Clinical and Laboratory Standards Institute
CN: Gentamicin
CPR: Carbapenemase producer Enterobacteriales
CRE: Carbapenem resistance Enterobacteriales
CRO: Ceftriaxone
CTX: Cefotaxime
CTX-M: CTX for cefotaximase and M for Munich
CXM: Cefuroxime
DNA: Deoxyribonucleic acid
*E. coli*: Escherichia coli
EDTA: Ethylene diamine tetraacetic acid
ESBL: Extended-Spectrum β-Lactamase
GNB: Gram-negative bacilli
ICU: Intensive care unit
IEND: Institute of Endemic Diseases
IMP: Imipenemase Metallo-β-lactamase
IPM: Imipenem
*K. pneumonia*: *Klebsiella pneumonia*
KPC: *Klebsiella pneumoniae* carbapenemase-producer
LMICs: Low and middle income countries
MBL: Metallo-β-lactamas
MDR: Multi drug resistance
MEM: Meropenem
MHT: Modified Hodge test
NDM: New Delhi Metallo-β-lactamase
OXA: oxacillinases
*P. aeruginosa*: *Pseudomonas aeruginosa*
PCR: Polymerase Chain Reaction
*S. maltophilia*: *Stenotrophomonas maltophilia*
SHV: Sulphydryl Reagent Variable
SPSS: Statistical Package for the Social Sciences
SUH: Soba University Hospital
SXT: Trimethoprim-sulfamethoxazole
TEM: Named after patient Temoniera
TEM: Temocillin
VIM: Verona integron-encoded Metallo-β-lactamase
WHO: World Health Organisation
°C: Celsius degree
16SrRNA: 16 small ribosomal RNA
μg: Microgram
